# The Effects Of Latent Classes Of Stress On Health Outcomes In Korean Middle-aged Adults: A Focus On Gender Differences

**DOI:** 10.1093/geroni/igab046.3165

**Published:** 2021-12-17

**Authors:** Kyuyoung Cho

**Affiliations:** Dong-A University, Busan, Pusan-jikhalsi, Republic of Korea

## Abstract

This study indicated the effect of the latent classes of stress on the physical and psychological health outcomes in Korea. Using the 2010 Korea Health Panel Study, 1,689 middle-aged adults (women: n=793, men: n=896) were analyzed to identify the latent classes of stress by gender using Latent Profile Analysis (LPA). After the determination of the number of latent classes, health outcomes (anxiety/depression and health status) were also regressed on the latent classes including covariates (age, marital status, and education level). The perceived stresses (financial diversity, disease of self or family, children's education, and family conflicts) are classified as the 2-class model for women and the 3-class model for men. The classes of women are named 'high stress and 'low stress; however, the classes of men are named 'family-related stress', 'disease stress', and 'low stress.' The different combinations of stress are associated with anxiety/depression and health status respectively. This study will discuss the difference of latent stress classes by gender and extend the understanding of stress groups and health outcomes.

